# Sugar-Sweetened Beverages and Childhood Obesity: A Life-Course Perspective

**DOI:** 10.3390/nu18193118

**Published:** 2026-09-22

**Authors:** Bianca Simionescu, Ancuța Lupu, Alexandra Sidoreac, Vasile Valeriu Lupu, Elena Roxana Buzilă, Raluca Olariu, Bogdan Novac, Ileana Ioniuc, Mihaela Monica Alexoae, Roxana Șerban, Otilia Novac, Emil Anton, Oana Raluca Temneanu

**Affiliations:** 1 Faculty of Medicine, Iuliu Hatieganu University of Medicine and Pharmacy, 400012 Cluj-Napoca, Romania; bianca.simionescu@umfcluj.ro; 2 Faculty of Medicine, Grigore T. Popa University of Medicine and Pharmacy, 700115 Iasi, Romania; alexandra.sidoreac@d.umfiasi.ro (A.S.); vasile.lupu@umfiasi.ro (V.V.L.); elena-roxana.buzila@umfiasi.ro (E.R.B.); raluca.olariu@umfiasi.ro (R.O.); bogdan.novac@umfiasi.ro (B.N.); ileana.ioniuc@umfiasi.ro (I.I.); mihaela.alexoae@umfiasi.ro (M.M.A.); roxana-n-serban@umfiasi.ro (R.Ș.); otilia.novac@umfiasi.ro (O.N.); emil.anton@umfiasi.ro (E.A.); temneanu.oana@umfiasi.ro (O.R.T.)

**Keywords:** sugar-sweetened beverages, adolescence, childhood obesity, early-life nutrition, dietary programming, fructose, visceral adiposity, insulin resistance, MASLD, food preference

## Abstract

**Background/Objectives**: Childhood obesity represents a major global health challenge. Sugar-sweetened beverages (SSBs) remain an important modifiable dietary exposure; however, adolescent beverage behavior is often examined independently of dietary patterns established earlier in life. This narrative review examines SSB consumption and adiposity across the life course, particularly during the first 1000 days as a sensitive period for developing dietary habits. **Methods**: Targeted literature searches were conducted in PubMed, Scopus, and Web of Science (January 2021–13 August 2026). We prioritized systematic reviews, meta-analyses, prospective cohort studies, randomized trials, and mechanistically informative studies. Study selection was purposive, and causal language was reserved for rigorous designs. **Results**: SSB intake is associated with higher body mass index, waist circumference, body fat percentage, and pediatric obesity. Potential mechanistic pathways include incomplete energy compensation for liquid calories, fructose-related hepatic de novo lipogenesis under conditions of high exposure, and insulin resistance; however, the independent contribution of fructose remains difficult to disentangle from excess energy intake, beverage form, and the broader dietary matrix. SSB consumption patterns may emerge early and track through childhood, influenced by family, socioeconomic, and commercial determinants. Evidence does not consistently support the hypothesis that repeated sweetness exposure increases subsequent sweetness liking. Metabolic consequences include dyslipidemia, hypertension, and metabolic dysfunction-associated steatotic liver disease (MASLD). Prevention approaches include family- and school-based interventions, beverage reformulation, and fiscal policies. **Conclusions**: A life-course perspective supports shaping beverage environments early, strengthening healthy home food environments, and implementing structural public-health interventions rather than addressing unhealthy patterns only after they become established. However, policy effects may vary across pediatric subgroups.

## 1. Introduction

Childhood and adolescent obesity has evolved from an individual clinical problem into a major global public health challenge. Its consequences extend beyond excess body weight and include early abnormalities in glucose metabolism, blood pressure, lipid homeostasis, hepatic fat accumulation, and vascular risk. The prevention and management of childhood obesity and its psychological and physical comorbidities therefore represents an urgent priority [1]. Within the broader obesogenic food environment, sugar-sweetened beverages (SSBs) are of particular interest because they deliver rapidly absorbable sugars in liquid form, provide energy with little accompanying nutrient value, and are widely available and heavily integrated into contemporary dietary patterns.

In this review, SSBs are the primary exposure of interest and refer to beverages containing added caloric sugars, including carbonated soft drinks, fruit-flavored drinks with added sugars, sports and energy drinks, and other sweetened beverage products. Related exposures are considered separately and are not treated as interchangeable with SSB intake. Added sugars and free sugars represent broader but distinct dietary constructs that may include sugar exposure from sources other than SSBs; fructose refers to a specific monosaccharide that may be consumed from both beverage and non-beverage sources; and sweetness exposure or preference represents a sensory and behavioral construct rather than a measure of sugar intake. One hundred percent fruit juice is not classified here as an SSB but is discussed separately because the sugars naturally present in whole fruit are released into the liquid matrix during juicing and are classified as free sugars, providing an informative comparison for potential beverage-form effects. Non-nutritively sweetened beverages are likewise treated as a distinct category and are considered primarily where beverage substitution is relevant. Data from 2018 indicated that SSB intake among children and adolescents remains substantial across the globe. An analysis covering children and adolescents aged 3–19 years across 185 countries estimated a mean global intake of approximately 3.6 standardized servings per week, with marked geographical and sociodemographic variation [2]. Importantly, global intake increased considerably between 1990 and 2018, indicating that SSB exposure is not confined to a limited number of high-income settings.

The association between SSB intake and adiposity is supported by multiple levels of evidence. A comprehensive review of the field concluded that cohort studies on clinical outcomes and clinical trials assessing cardiometabolic risk factors together support an etiological role for SSBs in weight gain and cardiometabolic disease [3]. Updated systematic review and meta-analytic data incorporating prospective cohorts and randomized interventions support a relationship between higher SSB consumption and increased body mass index (BMI) in both children and adults [4]. An umbrella review of existing meta-analyses similarly identified harmful associations between dietary sugar consumption and a broad range of endocrine, metabolic, and cardiovascular outcomes, while noting that the certainty of much of this evidence is moderate at best [5]. More recent population studies continue to identify associations between SSB consumption and childhood overweight or obesity, although the magnitude and interpretation of these relationships depend on study design, residual confounding, dietary measurement, and the extent to which total energy intake is considered.

However, considering SSB consumption only at the point when obesity emerges may overlook an important part of its developmental context. Adolescents do not encounter sweet beverages without pre-existing dietary habits. Beverage familiarity, feeding interactions, and food-related expectations begin to develop during infancy and early childhood. Emerging longitudinal evidence indicates that recognizable SSB consumption trajectories can already be identified during early life, while early exposure to sweetened beverages may be associated with less favorable dietary patterns later in life [6]. The first 1000 days represent a critical window in which nutritional exposures and early-life determinants can profoundly influence childhood obesity trajectories and subsequent adult health outcomes [7].

Recent evidence reinforces this perspective on the first 1000 days. A natural experiment based on the end of postwar sugar rationing in the United Kingdom found that lower sugar exposure during gestation and early childhood was associated with substantially lower risks of type 2 diabetes and hypertension in adulthood, suggesting that the timing of sugar exposure may have long-term biological and behavioral importance [8]. An overview of systematic reviews found limited, low-certainty evidence that early-life nutritional interventions prevent childhood obesity; only reduced infant-formula protein reached moderate certainty, while multicomponent interventions involving responsive feeding or physical activity were modestly beneficial [9].

The present review therefore proposes that adolescent SSB consumption and its association with adiposity should be understood within an evolving pediatric dietary trajectory. This review examines the pathway from early-life dietary exposure to persistent beverage behaviors; adolescent SSB intake; excess energy exposure and fructose-related metabolic effects; visceral and ectopic adiposity; and subsequent insulin resistance, dyslipidemia, hypertension, and MASLD. We also consider how interventions at the family, clinical, school, community, and policy levels may modify this trajectory.

## 2. Literature Search Strategy

This narrative review synthesizes recent evidence on early-life dietary programming, SSB consumption patterns, and metabolic consequences across the pediatric life course. Targeted literature searches were conducted in PubMed, Scopus, and Web of Science for peer-reviewed publications published from January 2021 onwards, with the final literature search performed on 13 August 2026, supplemented by backward citation searching and selected earlier foundational studies. Search concepts combined terms related to sugar-sweetened beverages, added and free sugars, fructose, infancy, the first 1000 days, childhood, adolescence, obesity and adiposity, visceral or hepatic fat, insulin resistance, MASLD, feeding practices, sweet taste preference, digital food marketing, school interventions, and public-health policy. Study selection was purposive and guided by relevance to the life-course framework of the review. Priority was given to systematic reviews and meta-analyses, prospective cohort studies, randomized controlled trials, major population studies, clinical guidance, and mechanistically informative studies. Within these categories, studies were prioritized when they provided longitudinal data, pediatric-specific outcomes, direct assessment of SSB or related beverage exposure, clinically relevant adiposity or metabolic outcomes, or evidence particularly informative for the developmental stages and mechanistic pathways addressed in the review. Cross-sectional studies were retained when they provided relevant contemporary population-level evidence or addressed outcomes for which stronger longitudinal or experimental pediatric evidence was limited.

Studies were considered eligible when they directly addressed pediatric SSB or related beverage exposure, early-life dietary exposures relevant to the proposed life-course framework, adiposity or cardiometabolic outcomes, developmental beverage patterns, or prevention and policy interventions relevant to children and adolescents. When multiple studies addressed a similar question, preference was given to pediatric-specific evidence, longitudinal or experimental designs, larger and well-characterized populations, direct assessment of beverage exposure, clinically relevant outcomes, and studies most informative for the developmental stages or mechanistic questions addressed in the review. Studies were excluded when they were outside the scope of the review, did not provide evidence relevant to the exposure–outcome relationships considered, or consisted of editorials, commentaries, case reports, or preprints. Grey literature was not systematically searched and was not used as primary evidence.

English-language peer-reviewed publications directly relevant to the scope of the review were considered; editorials, commentaries, case reports, preprints, and other grey literature were not used as primary evidence. Because this was a narrative rather than a systematic review, no formal risk-of-bias assessment or quantitative evidence synthesis was undertaken. Methodological features relevant to interpretation, including study design, temporality, potential confounding, and the distinction between observational and interventional evidence, were nevertheless considered when interpreting individual studies and formulating the synthesis. No validated study-quality or certainty-of-evidence instrument was applied. However, the key studies summarized in Table 1 were assigned a qualitative interpretive-confidence rating based on study design, temporality, sample size, exposure and outcome measurement, precision, and susceptibility to confounding. Causal language was reserved for evidence supported by randomized or otherwise strong causal designs, whereas observational findings were described as associations. Where the evidence base was internally contradictory, both directions of evidence were reported rather than selectively emphasizing findings consistent with the review’s framing.

## 3. Early-Life Origins of Beverage Behavior

### 3.1. The First 1000 Days as a Sensitive Nutritional Window

The first 1000 days, extending from conception through approximately the second year of life, represent a period of rapid metabolic, neurological, sensory, and behavioral development. Nutrition during this period not only influences somatic growth but also shapes metabolic responses and dietary behavior. Consequently, early nutritional exposures may affect subsequent health through both biological programming and learned patterns of food acceptance [20].

Maternal lifestyle during pregnancy, early nutrition, and the environment in which infants are raised are considered relevant factors for the prevention of childhood obesity and for establishing healthy dietary trajectories from the earliest periods of development [21].

The natural experiment created by the end of sugar rationing in the United Kingdom provides particularly compelling evidence for the relevance of timing. Zheng and colleagues, in a natural experiment using UK Biobank data, compared individuals with different durations of exposure to sugar rationing during gestation and early postnatal life. Exposure to sugar rationing throughout gestation and the first 1–2 postnatal years was associated with lower risks of several cardiovascular outcomes in adulthood; for example, hazard ratios (HRs) were 0.80 (95% confidence interval [CI]: 0.73–0.90) for cardiovascular disease and 0.76 (95% CI: 0.66–0.92) for atrial fibrillation compared with no exposure to rationing [22]. Although these findings do not demonstrate that early sugar exposure acts exclusively through later beverage preference or adiposity, they provide important support for the concept that early sugar exposure may have long-lasting health consequences through developmental programming mechanisms.

Early-life nutrition should therefore not be considered solely from the perspective of nutrient sufficiency. The type, concentration, frequency, and context of exposure may contribute to an infant’s developing relationship with food. The Developmental Origins of Health and Disease paradigm provides a framework for understanding how early-life environmental exposures, particularly during the periconceptional, fetal, and neonatal periods, can program future health outcomes through epigenetic and metabolic mechanisms [23].

### 3.2. Added Sugars During Infancy and Early Childhood

Recent pediatric data indicate that added-sugar exposure begins well before school age. Research examining added-sugar consumption in the first two years of life has reported associations between higher added-sugar intake and measures of abdominal adiposity as well as selected appetite- and eating-related behaviors. This work is especially relevant because it moves the discussion beyond total body weight and suggests that metabolic and behavioral correlates of sugar exposure may already be detectable during infancy and toddlerhood [24].

These observations do not establish a deterministic pathway from infant sugar intake to adolescent obesity. Early dietary exposures interact with breastfeeding, complementary feeding, parental diet, socioeconomic conditions, food accessibility, cultural practices, and the broader dietary pattern. Within this broader context, exposure to added sugars may also occur through sweetened drinks, making early childhood relevant not only to overall sugar intake but also to the emergence of beverage-consumption patterns. However, early added-sugar exposure should not itself be interpreted as evidence that later SSB habits are established. The possibility that early dietary patterns may contribute to later dietary and health trajectories provides a rationale for examining infancy and early childhood as potential windows for prevention, while the long-term effects of interventions initiated during this period remain incompletely established [25].

In clinical practice, one distinction is particularly important. Prevention does not require elimination of naturally occurring sugars from nutrient-dense foods such as intact fruit or unsweetened dairy products. Rather, the principal concern is repeated exposure to added or free sugars delivered through foods of low nutrient density and, particularly, through sweetened beverages, which deliver concentrated sugar with limited satiating capacity relative to many solid foods [13,26,27].

### 3.3. Sweet Preference: What the Evidence Does and Does Not Support

Humans exhibit an early biological preference for sweetness—an adaptive response to the fact that, historically, sweet-tasting foods signaled a reliable source of energy. It is widely assumed that repeated exposure to sweetened foods and beverages further amplifies this preference, producing a self-reinforcing “sweet tooth.” This assumption warrants explicit scrutiny, because it is not well supported by the current evidence.

An updated review of human intervention trials and prospective cohort studies found no consistent evidence that exposure to sweet foods or beverages increases subsequent general liking for sweetness in either adults or children [28]. However, the available studies vary in participant age, duration and type of exposure, and the methods used to assess sweet taste preference, limiting the strength of conclusions that can be drawn across populations and experimental settings [28]. Consistent with this, a cohort study of infants found no association between sweetness exposure between 3 and 12 months and sweetness liking at 6 or 12 months, despite a sharp increase in sweetness exposure across that period; sweetness liking at 6 months did, however, predict liking at 12 months [10].

This distinction matters for how prevention is justified. The case for limiting early SSB exposure does not depend on the claim that sweetness exposure recalibrates the palate. It rests instead on more robustly supported grounds: SSBs deliver discretionary energy and rapidly absorbable sugars that meet no nutritional requirement in infancy; early beverage patterns track into later childhood; and the household beverage environment determines what is habitually available. Dietary experience does shape familiarity, meal structure, and food-related expectations, and caregiver behavior determines not only what is available but also the emotional and social meaning attached to food [11,23]. A large individual participant data meta-analysis of parent-focused behavioral interventions found no effect on BMI z-score at age 24 months (mean difference −0.01, 95% CI: −0.08 to 0.05; high-certainty evidence), indicating that such interventions as currently designed are insufficient to prevent obesity by this age [29].

Accordingly, early-life sugar exposure should be conceptualized as one component within a broader food environment rather than as a single causal agent acting through taste programming. The clinically relevant question is therefore not whether exposure to sweetness permanently alters a child’s palate—a consistent effect has not been demonstrated in the available evidence—but whether a permissive beverage environment normalizes habitual consumption of products that contribute discretionary energy without nutritional benefit.

## 4. From Early Exposure to Persistent Beverage Patterns

### 4.1. Tracking of SSB Consumption

Longitudinal evidence from several independent cohorts indicates that beverage behaviors established in infancy and early childhood can show measurable continuity into later childhood, although the observed degree of tracking varies and does not imply behavioral determinism. Tracking was operationalized differently across studies, including trajectory-based classification of repeated SSB measurements, stability of intake categories across follow-up assessments, and prospective associations between early exposure and later consumption. Arora et al. identified distinct high- and low-SSB trajectories from infancy through 3 years of age, with trajectory membership associated with maternal and socioeconomic characteristics [30]. In the Norwegian Mother and Child Cohort Study, 9025 children were assessed at 18 months, 36 months, and 7 years; intake of SSBs showed fair-to-moderate tracking over childhood, and children classified into lower or higher intake groups at 18 months tended to remain in the corresponding groups at later assessments [31]. Similarly, in the US Infant Feeding Practices Study II, any SSB consumption during infancy was associated with more than twice the adjusted odds of daily SSB consumption at 6 years of age compared with no infant SSB exposure (aOR 2.22) [32]. Further evidence comes from the Canadian TARGet Kids! prospective cohort, in which 999 children were followed from early childhood (≤2.5 years) to 5–9 years of age. Early consumption of sugar-containing beverages was associated with greater odds of consumption later in childhood (adjusted OR 4.03, 95% CI 2.92–5.55), with a particularly strong association observed for soda and sweetened drinks [33]. Together, these findings support continuity of beverage behavior from infancy into school age across independent populations.

Evidence spanning the complete interval from infancy through adolescence is less extensive. Longitudinal cohorts at later ages nevertheless indicate that distinct SSB patterns can persist across adolescence: in the Raine Study, trajectories based on intake at 14, 17, and 20 years included consistently low, increasing, decreasing, and consistently high consumption, with the consistently high trajectory associated with greater DXA-measured fat mass at age 20 [34]. Available data therefore support tracking of SSB-related behaviors over portions of the pediatric life course, but relatively few cohorts have repeatedly measured SSB intake across the entire period from infancy through adolescence. The tracking component of the proposed life-course model should therefore be regarded as probabilistic rather than deterministic, and the full infancy-to-adolescence pathway remains incompletely characterized.

### 4.2. Family Feeding Practices and the Home Food Environment

The household represents the first, and arguably one of the most influential, food environments during childhood [35]. Before children can make independent purchasing decisions, caregivers largely determine beverage availability, accessibility, portion sizes, meal structure, and the types of drinks offered with meals or in response to thirst, distress, celebration, or reward. Within this environment, parental modeling may be particularly influential: a household in which water is routinely consumed and readily available establishes a markedly different beverage norm from one in which sweetened drinks are regularly purchased and consumed. The distinction between restriction and structure is also important. Highly controlling feeding practices may inadvertently increase the salience of restricted products, whereas structured availability and responsive feeding allow caregivers to shape the food environment while supporting children’s developing sensitivity to internal hunger and satiety cues.

Family-based prevention should therefore avoid framing childhood sugar consumption as resulting simply from inadequate parental discipline or individual choice. Caregiver feeding practices themselves operate within broader socioeconomic and environmental constraints, including household income, parental education, working conditions, food prices and accessibility, cultural norms, and pervasive food and beverage marketing. Recognizing these interacting influences supports a more comprehensive approach in which families are viewed not only as targets of nutritional education but also as participants within a wider food environment that can either facilitate or undermine healthy beverage choices. A scoping review of feeding practices in families experiencing food insecurity or socioeconomic disadvantage concluded that interventions promoting responsive feeding may not meet the needs of these households and that intervention design should be sensitive to poverty-related constraints [11].

### 4.3. Socioeconomic Context

Socioeconomic disadvantage repeatedly emerges as an important determinant of SSB consumption. In the early-life trajectory study by Arora et al., characteristics related to socioeconomic disadvantage were associated with less favorable beverage trajectories [30]. At the population level, SSB consumption also varies substantially according to geography, age, parental education, and other social characteristics [2].

Consequently, interventions directed exclusively at individual knowledge are unlikely to fully address disparities in consumption. Educational interventions may improve awareness, but their effectiveness remains constrained when healthier alternatives are less accessible, more expensive, or less aggressively promoted. Population-level interventions addressing structural determinants—including pricing, availability, and marketing—may be necessary to achieve equitable reductions in SSB consumption across socioeconomic groups [36].

## 5. Adolescence as a Critical Transition Period

Adolescence represents a transition from predominantly caregiver-controlled food exposure toward increasing dietary autonomy and greater exposure to environmental and commercial influences. These include school and retail food environments and increasingly prominent digital influences. Such factors represent broader features of the adolescent food environment and should not all be interpreted as independently established determinants of SSB consumption [36]. For SSBs specifically, evidence relating to school-based interventions and digital exposure is discussed below [37,38]. Against the background of the tracking evidence discussed in Section 4.1 [30,31,32,33,34], adolescence may represent a period in which previously established beverage patterns persist or are modified under conditions of greater autonomy and environmental exposure, rather than a stage in which such patterns necessarily emerge de novo. Children with previously established sweet-beverage consumption may subsequently encounter an environment in which those beverages become more readily available and socially embedded. In addition to soft drinks, adolescents encounter sweetened coffee products, sports beverages, energy drinks, flavored teas, and other products positioned as lifestyle products rather than as explicitly “sugary” beverages. Caffeinated energy drinks deserve separate mention: an overview of systematic reviews commissioned by the UK government reported that, worldwide, between 13% and 67% of children had consumed energy drinks in the past year, and that frequent consumption was associated with lower psychological, physical, educational, and overall well-being, although the authors graded the underlying evidence as weak and predominantly cross-sectional [39]. Given the predominantly cross-sectional nature of this evidence, the direction of the reported associations cannot be established, and residual confounding and reverse causality cannot be excluded.

The digital environment is a distinctive feature of contemporary adolescence and represents one domain for which evidence directly related to SSB consumption is available. A systematic review of social media exposure and diet in children and adolescents aged 2–18 years found that social media use was associated with skipping breakfast, higher intake of unhealthy snacks and sugar-sweetened beverages, and lower fruit and vegetable intake, independent of age; exposure to unhealthy digital food images was accompanied by increased activation in reward- and attention-related brain regions [37].

Adolescent behavior is also influenced by the clustering of health behaviors. Higher SSB intake may coexist with fast-food consumption, inadequate sleep, sedentary behavior, high screen exposure, and lower diet quality [40,41]. This clustering complicates causal interpretation in observational research but also indicates that SSB consumption may serve as a clinically useful marker of a broader obesogenic lifestyle.

Recent prospective data further suggest that high SSB consumption during adolescence may be associated with adverse trajectories beyond body weight, including physical health, sleep, and emotional health outcomes, emphasizing the need to evaluate SSB intake within a broader developmental context [42].

## 6. Epidemiological Evidence Linking SSB Intake to Pediatric Adiposity

The epidemiological association between SSB intake and adiposity is supported by cross-sectional, longitudinal, and interventional evidence, although the strength of causal inference varies substantially across study designs. Cross-sectional studies are useful for characterizing contemporary associations but cannot establish temporality, whereas prospective cohort studies provide stronger evidence regarding temporal relationships while remaining susceptible to residual confounding and exposure-measurement error. Randomized interventions can provide stronger causal evidence, although their duration, feasibility, and specific intervention design may limit direct inference regarding long-term habitual SSB consumption and adiposity.

Recent cross-sectional evidence remains informative for characterizing SSB consumption and adiposity within contemporary pediatric populations. Li et al. surveyed 3402 primary and secondary school students aged 7–17 years in Beijing, China, among whom the overall prevalence of overweight or obesity was 38.5% [43]. After adjustment for relevant covariates, participants consuming SSBs ≥ 7 times per week had higher odds of overweight or obesity than those consuming SSBs < 1 time per week (odds ratio [OR] = 1.40, 95% CI: 1.09–1.78). Similarly, an SSB intake of ≥500 mL/day was associated with higher odds of overweight or obesity compared with an intake of <150 mL/day (OR = 1.32, 95% CI: 1.09–1.59) [43]. These findings provide recent population-level evidence of a positive gradient between SSB consumption and excess weight in school-age children and adolescents. Nevertheless, because exposure and outcome were measured concurrently, temporal direction cannot be established, and residual confounding remains possible.

The consistency of this association across very different settings is notable. A cross-sectional analysis pooling data from 405,528 school-going adolescents across 107 countries and regions found a positive correlation between the prevalence of daily soft drink consumption and the prevalence of overweight and obesity (partial correlation coefficient 0.44; 95% CI: 0.26–0.58; *p* < 0.001). At the individual level, daily soft drink consumption was associated with a higher odds ratio of overweight or obesity (OR 1.14, 95% CI: 1.08–1.21) for daily versus non-daily consumption [12]. Because most of the countries included were low- and middle-income, these data indicate that the association is not confined to high-income food environments.

Prospective evidence provides stronger support for temporality. An updated systematic review and meta-analysis by Nguyen et al., incorporating cohort studies and randomized controlled trials, concluded that SSB consumption was associated with greater BMI and body weight in children and adults [4]. Such evidence is important because it demonstrates consistency across designs and populations rather than relying on isolated observational associations.

The outcomes assessed should also extend beyond BMI. BMI is practical and clinically useful but cannot distinguish lean from fat mass or characterize fat distribution. A dose–response meta-analysis of 33 studies including 121,282 children and adolescents found that high SSB intake was associated not only with a 0.75 kg/m^2^ higher BMI (95% CI: 0.35–1.15) but also with a 2.35 cm larger waist circumference (95% CI: 1.34–3.37) and a 2.81 percentage-point higher body fat percentage (95% CI: 2.21–3.41), with no departure from linearity [13]. Central and visceral adiposity are metabolically important because visceral adipose tissue is closely related to insulin resistance, dyslipidemia, inflammation, and ectopic lipid deposition; evidence linking sweetened beverage intake with these compartments therefore strengthens the biological plausibility of associations observed using BMI alone.

The interpretation of beverage-adiposity associations also requires consideration of what SSBs replace. Observational associations between low- and no-calorie sweetened beverages and cardiometabolic outcomes require cautious interpretation because reverse causation and residual confounding are important concerns. Analyses that explicitly model beverage substitution do not consistently indicate cardiometabolic harm and suggest that replacing SSBs with low- or no-calorie alternatives may confer benefit, although the certainty of the evidence remains limited [26].

## 7. Mechanisms Linking Sugar-Sweetened Beverages to Adiposity and Metabolic Dysfunction

### 7.1. Liquid Calories and Incomplete Energy Compensation

A central feature of SSBs is not simply their sugar content but their delivery of energy in liquid form. Energy consumed in beverage form may be incompletely compensated for at subsequent eating occasions. In a double-blind pediatric randomized trial, sugar-sweetened and sugar-free beverages produced similar satiety despite their differing energy content, a finding consistent with incomplete compensation for beverage-derived energy [27]. This provides a plausible pathway through which habitual SSB intake may increase total energy intake [44]. A pediatric narrative review identified decreased satiety and incomplete compensatory reduction in energy intake at subsequent meals as a leading proposed mechanism underlying the association between SSB intake and weight gain in children, alongside effects on the gut microbiota and on eating behaviors [45].

Over time, repeated incomplete compensation for beverage-derived energy could contribute to positive energy imbalance [45].

### 7.2. Sugar Composition and Glycemic Exposure

The type and concentration of added sugars in SSBs vary across beverage categories, product formulations, manufacturers, and countries. Depending on the product and market, SSBs may contain sucrose, high-fructose corn syrup, glucose–fructose mixtures, or other caloric sweeteners. Sucrose is a disaccharide of glucose and fructose in equal molar proportions. The glucose component contributes to glycemic and insulin responses, whereas fructose is metabolized through distinct intestinal and hepatic pathways that are relevant to hepatic lipid metabolism and uric acid production, particularly at high exposures [46].

The biological effect of an SSB therefore cannot be reduced to one mechanism. Its obesogenic potential may reflect the combination of energy content, limited satiety, repeated glycemic exposure, sugar composition, large portion sizes, and consumption frequency.

### 7.3. Fructose Metabolism and Hepatic De Novo Lipogenesis

The metabolic handling of fructose provides a biologically plausible mechanistic link between SSB intake and metabolic dysfunction. Following absorption, substantial first-pass splanchnic extraction of fructose occurs in humans. Isotope-tracing studies in humans and experimental animals indicate that the intestine can metabolize a substantial fraction of dietary fructose before hepatic exposure, although the relative intestinal and hepatic contributions at usual pediatric intakes remain uncertain [47,48]. Hepatic fructose phosphorylation is catalyzed by ketohexokinase, generating fructose-1-phosphate and facilitating rapid substrate flux into downstream pathways. Unlike glucose, fructose enters glycolytic pathways downstream of the major phosphofructokinase regulatory step. Under conditions of high substrate availability, fructose-derived carbon can therefore provide substrate for hepatic de novo lipogenesis; whether this translates into pathological lipid accumulation in children depends on dose, energy balance, and metabolic context [49].

When hepatic substrate availability exceeds immediate oxidative or glycogen-storage requirements, carbon derived from fructose can contribute to de novo lipogenesis and triglyceride synthesis. Under sustained high-substrate conditions, this pathway may favor hepatic triglyceride synthesis and lipid accumulation, although the magnitude of these effects at usual pediatric intakes remains uncertain. In experimental animal models, high fructose exposure has been shown to alter intestinal glucose handling and to promote hepatic lipid accumulation and insulin resistance [50]. These model-based findings should not be directly extrapolated to usual pediatric dietary exposures.

Fructose phosphorylation also consumes adenosine triphosphate (ATP). High metabolic flux may increase adenosine monophosphate (AMP) degradation and uric acid generation, while oxidative and mitochondrial stress have been proposed as additional mechanisms potentially contributing to metabolic dysfunction. The hypothesized fructose–methylglyoxal–AMP-activated protein kinase (AMPK) axis is one proposed mechanistic framework, but its importance in human disease has not been established. These mechanisms and their pediatric implications were previously synthesized by Luca et al. in a critical review of fructose-related metabolic risk in children [51].

Importantly, these mechanisms should not be interpreted as evidence that fructose itself is an established independent driver of SSB-related metabolic dysfunction or that all dietary fructose is intrinsically harmful at physiological intakes. The metabolic context and food matrix matter profoundly. Fructose contained within intact fruit is delivered together with fiber, water, micronutrients, and an intact cellular matrix, and at substantially different rates and quantities from fructose delivered in a large sweetened beverage. Moreover, epidemiological evidence does not consistently separate fructose-specific effects from those associated with beverage form, overall energy exposure, or the broader dietary matrix [3,45,51]. Fructose-related pathways should therefore be regarded as biologically plausible contributors to SSB-associated metabolic effects rather than as independently established causal mechanisms. The mechanistic concern considered here relates primarily to concentrated, rapidly consumed sugar exposure in the context of dietary excess rather than to fructose-containing whole fruit.

### 7.4. Visceral and Ectopic Fat Accumulation

Cardiometabolic risk is not captured by total weight gain alone. Visceral adipose tissue and ectopic hepatic fat are strongly associated with insulin resistance and cardiometabolic risk. This distinction is particularly relevant in children because individuals with similar BMI values may have substantially different metabolic phenotypes.

Longitudinal evidence suggests that higher sweetened-beverage intake may be associated with central as well as overall fat accumulation, although direct imaging-based evidence remains limited. In ALSPAC, increasing SSB consumption between 10 and 13 years was associated with greater waist circumference at 13 years among children with plausible dietary reporting, including after adjustment for BMI and total fat mass [52]. Similarly, in the European Youth Heart Study, consuming more than one SSB serving per day at 15 years was associated with a greater increase in waist circumference over the subsequent 6 years [53]. Adolescent SSB trajectories in the Raine Study were also related to DXA-measured total fat mass at age 20, although fat distribution was not specifically assessed [34].

More detailed compartment-specific longitudinal evidence comes from Project Viva. Higher fruit-juice intake at 1 year of age was associated with greater DXA-derived visceral adipose tissue in mid-childhood and early adolescence, with a stronger association for visceral than subcutaneous abdominal fat [14]. Because this exposure was 100% fruit juice rather than SSB, and comparable longitudinal VAT measurements have not yet been widely replicated in independent pediatric cohorts, the finding should be interpreted as supportive of the proposed pathway rather than definitive evidence of SSB-specific visceral-fat programming.

SSB intake may therefore be relevant to both overall adiposity and its distribution, but the evidence is not equally strong for these outcomes. Longitudinal studies support associations with total and central adiposity, whereas evidence for preferential visceral or ectopic fat deposition remains comparatively limited. This distinction is also relevant to MASLD, in which abdominal adiposity, insulin resistance, and dietary exposures may converge through several pathways [54]. At present, the visceral-fat component of the proposed life-course model should therefore be considered biologically plausible but provisional.

## 8. Beyond Adiposity: Metabolic Consequences

### 8.1. Insulin Resistance

Insulin resistance is closely interrelated with central and hepatic fat accumulation, while direct metabolic effects attributable specifically to high sugar or fructose exposure remain less certain in children. Pediatric intervention studies provide mechanistic support, but their findings are not uniform. In a 9-day single-arm feeding intervention involving 41 children with obesity and high habitual sugar intake, dietary fructose was substantially reduced while starch was substituted to maintain the intended energy and macronutrient composition. The intervention was followed by reductions in hepatic de novo lipogenesis, liver fat, and visceral adipose tissue, together with improved insulin kinetics [55]. However, the absence of a concurrent control group and the short duration of the intervention limit attribution of these changes specifically to fructose.

Additional experimental evidence supports metabolic effects of reducing dietary sugars in selected high-risk pediatric populations, without establishing a fructose-specific mechanism. In an 8-week randomized controlled trial involving 29 adolescent boys with fatty liver disease, a diet low in free sugars reduced hepatic de novo lipogenesis and fasting insulin compared with the usual diet, alongside a greater reduction in hepatic fat [56]. Conversely, a small randomized crossover feeding study of six adolescents with obesity did not detect changes in insulin sensitivity or secretion after 7 days of a higher-fructose isocaloric diet compared with a lower-fructose diet [57]. Given the small sample and short exposure period, this null finding does not exclude fructose-specific effects. It does, however, illustrate that such effects are not consistently demonstrable under short-term isocaloric conditions.

Against this experimental background, Project Viva provides complementary longitudinal evidence using causal-inference methods applied to observational data. Using inverse-probability-weighted marginal structural models, Harnois-Leblanc et al. estimated a mean HOMA-IR difference of −0.28 units (95% CI, −0.61 to 0.02) in males under a hypothetical intervention limiting SSB intake to one serving per week throughout childhood compared with no intervention; the corresponding estimate in females was near the null (0.07 units; 95% CI, −0.13 to 0.29) [15]. These sex-specific estimates were small and imprecise, and should therefore be interpreted as supportive rather than definitive evidence of a possible benefit of lower childhood SSB exposure for later insulin resistance.

The clinical relevance of insulin resistance extends beyond overt type 2 diabetes. Compensatory hyperinsulinemia and reduced insulin sensitivity may precede dysglycemia, creating a period of subclinical metabolic dysfunction before diagnostic thresholds for diabetes are reached. Current pediatric evidence, however, does not support a simple model in which fructose exposure alone uniformly drives insulin resistance. A more cautious interpretation is that SSB-related metabolic risk may emerge through interacting effects of energy balance, adiposity, hepatic metabolism, and the broader dietary context.

### 8.2. Dyslipidemia and Cardiovascular Risk

Increased hepatic lipid synthesis and very-low-density lipoprotein export are mechanistically linked to elevated circulating triglycerides and an atherogenic lipid phenotype. A dose–response meta-analysis of cross-sectional studies in children and youth found that high SSB consumption was associated with a 1.21 mg/dL higher low-density lipoprotein cholesterol (95% CI: 0.23–2.20) and a 1.46 mg/dL lower high-density lipoprotein cholesterol (95% CI: −2.25 to −0.67), with evidence of non-linearity for LDL cholesterol [58]. The magnitude of these differences is small and the included studies were cross-sectional, so they should be interpreted as evidence of population-level association rather than as establishing a clinically meaningful lipid effect. Prospective data provide stronger evidence for temporal ordering, although not for causality. In the Raine Study, changes in SSB intake during adolescence were prospectively associated with changes in cardiometabolic risk factors, including triglycerides; several associations were attenuated after accounting for broader dietary patterns [59].

Clinically, adolescents with obesity and high SSB intake may present not merely with excess weight but with a clustering of elevated blood pressure, dyslipidemia, insulin resistance, central adiposity, and hepatic steatosis.

### 8.3. Hypertension and Uric Acid

Fructose-related uric acid generation represents one proposed pathway linking SSB intake with blood-pressure regulation, but current evidence does not establish that this pathway operates independently of beverage form, energy intake, or the broader dietary matrix. Mechanistically, high fructose flux can increase uric acid generation through ATP utilization and purine degradation; however, downstream roles for uric acid, endothelial dysfunction, and related blood-pressure pathways are supported predominantly by preclinical and adult evidence, with less direct evidence in children. More broadly, the association between early-life sugar rationing and lower cardiovascular risk observed in the UK natural experiment is particularly relevant because it suggests that cardiometabolic consequences may emerge long after the exposure window [22].

More direct pediatric experimental evidence remains limited. In a randomized crossover trial of 30 overweight or obese adolescent boys who habitually consumed SSBs, three weeks of reduced-fat milk resulted in lower systolic blood-pressure z-scores and serum uric acid than an energy-equivalent sugar-sweetened soda phase [60]. Because the intervention changed the entire beverage matrix rather than fructose alone, these findings do not establish a fructose-specific or uric-acid-mediated blood-pressure mechanism.

Long-term prospective data also associate beverage intake from childhood onwards with incident hypertension in adulthood. In the Growing Up Today Study, 25,749 participants enrolled at a mean age of 12 years were followed for up to 25 years. Participants with the highest SSB intake (≥2 servings/day versus <3 servings/week) had a higher risk of incident hypertension (HR 1.52; 95% CI: 1.27–1.83), as did those with the highest fruit juice intake (HR 1.35; 95% CI: 1.06–1.71), whereas whole fruit intake was not associated with hypertension. In modeled substitution analyses, replacing one daily serving of SSB with milk, water, or whole fruit was associated with 13%, 9%, and 22% lower risk, respectively [16]. Notably, total fructose intake per se was not associated with incident hypertension, a pattern suggesting that the observed associations may depend on beverage form and food matrix rather than total fructose intake alone. Pediatric observational findings are also not uniform. Cross-sectional NHANES data associated greater SSB consumption with higher serum uric acid and systolic blood pressure among adolescents [61], whereas a cross-sectional analysis in the Raine Study found no direct association between dietary fructose intake and blood pressure, despite sex-specific associations involving serum uric acid [62]. Taken together, mechanistic studies implicating uric acid, oxidative stress, and inflammatory pathways in fructose-related blood-pressure regulation derive largely from animal models and adult populations; current pediatric human evidence supports biological plausibility but does not establish a confirmed uric-acid-mediated causal pathway.

### 8.4. Metabolic Dysfunction-Associated Steatotic Liver Disease

MASLD has become one of the most clinically relevant metabolic complications of pediatric obesity. Hepatic de novo lipogenesis, insulin resistance, adipose tissue dysfunction, and altered metabolic substrate delivery are central features of the pathophysiology of hepatic steatosis. MASLD is strongly associated with obesity and metabolic dysfunction, and its population burden has increased alongside the global rise in obesity and type 2 diabetes [63].

The relation between fructose exposure and pediatric MASLD remains incompletely defined. In pediatric obesity, higher fructose intake and unhealthy dietary patterns have been associated cross-sectionally with MASLD and adverse liver-related measures; however, these observational data do not establish progression to metabolic dysfunction-associated steatohepatitis (MASH) [64]. Despite evidence linking added sugars to metabolic syndrome, fructose’s impact on liver disease in youth remains incompletely understood.

Experimental evidence is stronger for free-sugar restriction than for fructose or SSBs specifically. In an 8-week randomized controlled trial of adolescent boys with fatty liver disease, a low-free-sugar diet reduced hepatic steatosis compared with the usual diet [65]. A mechanistic analysis within the same trial population also demonstrated lower hepatic de novo lipogenesis and fasting insulin with the low-free-sugar intervention [56]. These findings support a short-term causal effect of the low-free-sugar dietary intervention in this selected population but do not isolate SSBs or fructose as the sole active component.

Current pediatric recommendations recognize obesity, insulin resistance, dyslipidemia, and adverse dietary patterns as important components of MASLD risk, with lifestyle modification remaining fundamental to prevention and management [64]. The pathophysiology of MASLD is characterized by complex crosstalk between the liver and adipose tissue, including altered lipid flux, insulin signaling, inflammation, and metabolic substrate handling [66].

Within this framework, SSB intake represents a practical target because it is a modifiable source of discretionary energy and rapidly absorbed sugars. Nutrients and bioactive compounds have been explored as strategies to mitigate MASLD and MASH progression, but limiting excess SSB intake remains a practical component of lifestyle-based prevention and management [67,68].

## 9. Are All Sweet-Tasting Beverages Metabolically Equivalent?

The classification of beverages deserves careful consideration because sweetness alone does not determine metabolic effect.

One particularly important distinction concerns 100% fruit juice. Although fruit juice contains vitamins and bioactive compounds and does not necessarily contain added sugar, juicing removes much of the structural food matrix associated with whole fruit and permits rapid consumption of substantial quantities of naturally occurring free sugars. A 2024 systematic review and meta-analysis by Nguyen et al. found that each additional daily serving of 100% fruit juice was associated with a small increase in BMI among children in prospective cohort studies (0.03 BMI units, 95% CI: 0.01–0.05) [69]. Additional findings discussed in this review include associations between infant fruit-juice intake and greater visceral adiposity later in childhood and adolescence in Project Viva [14], and between higher fruit-juice intake and incident hypertension in the Growing Up Today Study, in which whole-fruit intake showed no corresponding association [16]. However, these findings should not be interpreted as fully independent corroboration across outcomes, because the same longitudinal cohorts contribute evidence to multiple sections of this review. In particular, Project Viva informs several analyses of childhood beverage exposure and later adiposity or metabolic outcomes, while the Growing Up Today Study contributes the long-term hypertension findings discussed above. This overlap limits the breadth of independent cohort evidence underlying some components of the proposed life-course model and should be considered when interpreting apparent consistency across metabolic endpoints. Nevertheless, the available evidence supports distinguishing fruit juice from whole fruit rather than assuming nutritional equivalence.

Artificially or non-nutritively sweetened beverages present a different challenge. Replacing an SSB with a no-calorie beverage can reduce sugar and energy exposure, but observational associations between artificially sweetened beverage consumption and adiposity may be influenced by reverse causation, as individuals at higher metabolic risk may preferentially choose “diet” products. Consequently, water should remain the preferred default replacement beverage, while no-calorie sweetened beverages should not be assumed to be metabolically equivalent either to SSBs or to water.

The clinical message is therefore not that every sweet-tasting beverage exerts the same effect, but that beverages must be evaluated individually. Energy content, sugar concentration and composition, food matrix, portion size, habitual frequency, and the beverage being displaced must all be considered.

## 10. Prevention Across the Pediatric Life Course

### 10.1. Infancy and Complementary Feeding

Because beverage behavior develops progressively, prevention should begin before SSB intake becomes habitual. During infancy and complementary feeding, the goal is not to create rigid dietary restriction but to establish a food environment in which minimally processed foods, diverse flavors, and unsweetened beverages are normal. The Healthy Life Trajectories Initiative (HeLTI) provides an intergenerational life-course framework for testing integrated interventions beginning before or during pregnancy and continuing through infancy and early childhood, with the aim of improving early adiposity and obesity-related trajectories [25].

Avoiding routine exposure to SSBs during the first two years may help limit unnecessary added-sugar exposure during a period in which dietary and beverage patterns are developing. Recent evidence associating early added-sugar intake with abdominal adiposity and eating behavior reinforces this preventive rationale [24]. Evidence from early-childhood obesity-prevention research includes interventions targeting several distinct domains, including dietary intake, responsive feeding practices, and physical activity. These components should not be assumed to have equivalent effects, and the magnitude and certainty of benefit vary according to intervention type and outcome assessed [9,29,70].

### 10.2. Early Childhood and the Family Environment

As children become increasingly autonomous, what is available in the household becomes central. Current pediatric guidance supports water as a habitual beverage and recommends limiting sugar-sweetened beverages, while whole fruit should generally be preferred to fruit juice [71,72]. Within the home environment, parental modeling and avoidance of routinely using sweetened beverages as rewards may further support a lower-sugar dietary environment [35].

Interventions should also preserve responsive feeding. Parents can determine which foods and beverages are available without coercively controlling how much a child consumes. This balance is important because prevention should promote healthy self-regulation rather than anxiety or moralization around food.

### 10.3. Adolescence

Adolescence requires a different approach. Simple parental restriction becomes less effective as independent purchasing grows. Counseling should therefore incorporate beverage literacy, understanding of portion sizes and hidden sugars, realistic substitution strategies, advice on sleep and physical activity, and the recognition of sports and energy drinks as potential major sugar sources. Energy drinks warrant specific attention in clinical encounters: a review of reported adverse health events in minors found that cardiovascular and neuropsychiatric systems were most frequently affected, and that events often occurred in the presence of additional trigger factors or pre-existing health conditions [73]. Because frequent energy drink use clusters with other health-compromising behaviors [39], asking about it can serve as an efficient screening question.

Healthcare professionals can use beverage intake as a brief screening marker during routine consultations. Asking what an adolescent drinks during meals, at school, during sports, and while socializing may reveal a substantial source of discretionary energy that is otherwise overlooked.

### 10.4. School-Based Approaches

Schools provide a major opportunity because they influence availability, education, peer norms, and water access simultaneously. A 2025 systematic review and meta-analysis evaluating school-based approaches to reducing sugar and SSB intake found that school interventions can contribute to reductions in sugar and SSB intake, supporting the inclusion of schools within multilevel prevention strategies [38].

Educational interventions are likely to be more effective when accompanied by environmental change, although effects vary across intervention designs and settings [38]. Teaching children to avoid SSBs while selling or prominently displaying them within the same setting creates a contradictory health environment. Chile’s experience is instructive here because school sales bans were implemented simultaneously with front-of-package warning labels and marketing restrictions rather than in isolation [17]. More rigorous evaluation and consistent reporting are needed to determine which combinations of school, family, community, and policy interventions are most effective and equitable.

## 11. Public Health and Policy Implications

Individual-level counseling can contribute to reducing SSB consumption, although its effectiveness is likely to vary according to intervention intensity, duration, population, and context. Because beverage behavior is also shaped by price, advertising, product formulation, availability, portion size, social norms, and default choices, clinical approaches may be complemented by upstream strategies addressing these broader environmental determinants [36].

SSB taxation has become one of the most widely discussed policy tools. A systematic review and meta-analysis of implemented SSB taxes worldwide found an overall tax pass-through rate of 82% (95% CI: 66–98%) and a mean reduction in SSB sales of 15% (95% CI: −20% to −9%), with no evidence of substitution to untaxed beverages [18]. Tiered levies can additionally stimulate industry reformulation.

The magnitude of the effect on childhood obesity itself is not uniform across jurisdictions, and here, the UK Soft Drinks Industry Levy (SDIL) provides the most direct evidence available. An interrupted time series analysis of the National Child Measurement Programme, covering over one million children annually, found an absolute reduction in obesity prevalence of 1.6 percentage points (95% CI: 1.1–2.1) among girls aged 10–11 years 19 months after implementation, with the greatest reductions in the two most deprived quintiles; no change was observed in boys of the same age or in children aged 4–5 years [19]. This sex- and age-specific pattern should be reported accurately rather than generalized: the levy was not associated with reduced obesity across the whole child population. Complementary modeling work projected that the observed sugar reductions would translate into fewer dental caries and fewer children classified as overweight or obese over ten years, with the largest gains in the most deprived areas and a small but significant narrowing of the slope index of inequality in life expectancy [74]. Fiscal measures can therefore operate at a population scale and complement individual-level approaches, while falling well short of solving the problem alone.

These findings should also be interpreted in light of the interrupted time-series design. Although this approach can estimate changes relative to pre-intervention trends at the population level, it does not provide the same protection against confounding as randomization. Concurrent secular changes, other public-health initiatives, changes in the food environment, or deviations from projected pre-levy trends may have contributed to the observed differences, and attribution of the changes exclusively to the SDIL is therefore not possible [19].

Product reformulation represents a complementary strategy because it reduces sugar exposure without requiring each consumer to make an active behavioral decision. Front-of-package warning labels may further improve recognition of high-sugar beverages, particularly when labels are simple and interpretable. Chile implemented mandatory front-of-package warning labels together with marketing restrictions and school sales bans; in the first phase, overall calories purchased declined by 3.5% and sugar purchased by 10.2% relative to a counterfactual based on pre-policy trends [17], and reductions were maintained or strengthened three years after implementation, with similar effects across socioeconomic strata [75].

Restrictions on marketing to children and adolescents are also important. Young consumers are exposed to commercial messaging during a developmental period characterized by increasing independence but incomplete maturation of decision-making processes. Digital marketing is particularly challenging because it blurs the boundaries between entertainment, peer endorsement, influencer content, and conventional advertising. A systematic review of marketing through social media and advergaming found significant effects on children’s pester behaviors, food choice, and food intake, although most included studies carried a high risk of bias [76]; the association between social media exposure and higher SSB intake in children and adolescents has been documented independently [37].

From an equity perspective, structural interventions may also reduce the extent to which health depends on individual resources. The association between socioeconomic circumstances and SSB trajectories indicates that dietary behavior is partly structured by the environments in which families make decisions [30], and the SDIL and Chilean evaluations both suggest that well-designed structural measures need not widen inequalities and may narrow them [19,74].

The most effective model is therefore likely to be layered: healthy beverage norms from infancy, caregiver support in early childhood, school-based environmental changes, adolescent education and clinical counseling, and pricing, marketing, labeling, and reformulation policies. Table 1 summarizes selected studies illustrating different components of the proposed life-course framework. The studies were selected for their relevance to distinct stages, pathways, and intervention levels within the framework and to reflect variation in study design, population, exposure, and outcome; they should not be interpreted as an exhaustive selection of the highest-certainty evidence identified in the review. Given their substantial methodological heterogeneity, a qualitative interpretive-confidence rating is provided to make differences in inferential strength more transparent.

Interpretive confidence was assigned qualitatively according to study design, temporality, sample size, exposure and outcome measurement, precision, directness to the question addressed, and susceptibility to confounding. Moderate confidence indicates evidence with important but non-fatal limitations; low confidence indicates evidence substantially limited by design, indirectness, heterogeneity, or inability to establish temporality. Among the studies selected for inclusion in Table 1, none was rated as high interpretive confidence because each retained at least one important limitation affecting causal or clinical interpretation. This assessment applies only to the studies summarized in Table 1 and should not be interpreted as a rating of the broader evidence base cited throughout the review. These ratings do not represent a formal risk-of-bias or certainty-of-evidence assessment.

## 12. Discussion

Despite extensive research on SSBs and obesity, several questions remain unresolved.

First, few studies follow the same participants from infancy through adolescence with sufficiently detailed repeated measurements of sugar exposure, beverage intake, caregiver feeding behavior, and body composition. Prospective longitudinal cohorts with repeated, age-specific assessment of beverage exposure from infancy through adolescence, detailed measurement of family dietary patterns and socioeconomic and environmental confounders, and repeated assessment of adiposity and metabolic outcomes are needed to determine whether early beverage exposure independently predicts later SSB consumption and cardiometabolic outcomes. Where feasible, intervention studies that modify beverage exposure early in life and include sufficiently long-term follow-up would provide stronger evidence regarding whether early exposure itself contributes to later beverage behavior and health outcomes. Second, the constructs in this field require more precise measurement and clearer separation. Exposure to sweetness, preference for sweetness, habitual SSB intake, reward sensitivity, and overall diet quality are related but distinct, and the evidence reviewed in Section 3.3 indicates that they do not move together in the way often assumed. Studies that measure sweetness liking directly, rather than inferring it from intake, are needed.

Third, future pediatric studies should move beyond BMI alone. Waist circumference, visceral adipose tissue, hepatic fat, insulin sensitivity, lipid phenotypes, and longitudinal metabolic biomarkers may identify clinically meaningful effects that BMI cannot detect.

Fourth, an important unresolved question is whether the metabolic associations attributed to fructose reflect fructose-specific effects or, at least in part, the form and dietary context in which fructose is consumed. The observation that total fructose intake was not associated with incident hypertension, whereas SSB and fruit juice intake were, suggests that beverage form and food matrix may modify the metabolic consequences of fructose-containing foods. Accordingly, the fructose-related pathways discussed in this review should be interpreted as biologically plausible components of SSB-related metabolic dysfunction rather than as independently established causal mechanisms. Future pediatric studies should distinguish effects attributable to fructose itself from those mediated by excess energy intake, liquid delivery, adiposity, and the broader dietary matrix.

Fifth, beverage substitution deserves greater attention. The health effect of reducing SSB consumption depends in part on what replaces them. Substitution with water may have different consequences from substitution with fruit juice, milk, or non-nutritively sweetened beverages.

Sixth, policy evaluation should extend beyond purchasing and sales outcomes to anthropometric and metabolic endpoints, and should report effects by sex, age, and socioeconomic position rather than in aggregate, since the SDIL evaluation demonstrates that pooled estimates can conceal substantial heterogeneity [19]. The absence of a detectable reduction in obesity among boys and reception-age children further indicates that population-level policy effects should not be assumed to be uniform across pediatric subgroups. Anthropometric and metabolic outcomes may, however, require substantially longer follow-up than purchasing or consumption indicators to detect meaningful population-level changes, and such measurements may not be routinely available in large-scale policy evaluations. Policy studies should therefore incorporate these outcomes where feasible while recognizing the complementary value of shorter-term behavioral, purchasing, and consumption endpoints.

An additional limitation concerns the restriction of the literature search to English-language publications. This may have excluded relevant evidence on school-based, fiscal, labeling, and other population-level interventions reported in other languages, including studies from regions in which such policies have been extensively implemented. Consequently, the policy evidence synthesized here may not fully represent the global intervention literature, and conclusions drawn largely from the UK, Chilean, and other English-language evidence should not be assumed to generalize uniformly across different policy, cultural, and socioeconomic contexts.

Finally, intervention research should examine whether the greatest benefit is obtained by targeting adolescents with established high intake or by preventing habitual SSB intake earlier in childhood. This distinction has major implications for pediatric counseling and public-health resource allocation.

An important remaining question is therefore how early in the life course trajectories associated with sustained SSB consumption become identifiable and potentially modifiable.

## 13. Conclusions

Sugar-sweetened beverage intake in adolescence should not be regarded as an isolated dietary behavior that begins when young people acquire purchasing independence. The evidence reviewed here is consistent with a broader developmental perspective in which early exposure to added sugars, caregiver feeding practices, socioeconomic circumstances, and the home food environment may contribute to dietary trajectories extending into later childhood and adolescence. However, because the evidence base was purposively selected within a narrative review and was not subjected to formal risk-of-bias assessment, the proposed life-course model should be interpreted as an integrative framework rather than as a systematically weighted causal model. These conclusions should also be interpreted in light of the heterogeneity of the included study designs, exposures, outcomes, and follow-up periods, as well as the limited number of studies spanning the full period from infancy to adolescence. Notably, this model does not require the common assumption that sweetness exposure itself heightens sweet preference, an effect that has not been consistently demonstrated in current intervention and cohort evidence. Once such patterns are established, the increasingly autonomous and commercially influenced context of adolescence means that high SSB consumption can contribute to positive energy balance while delivering large quantities of rapidly absorbable sugars. The metabolic consequences extend beyond weight gain and may involve interacting pathways related to hepatic substrate metabolism, de novo lipogenesis, adipose-tissue distribution, insulin resistance, dyslipidemia, blood-pressure regulation, and MASLD. Fructose-related mechanisms may contribute to these effects, particularly under conditions of high exposure, but their independent contribution remains difficult to disentangle from beverage form, total energy intake, and the broader dietary matrix. The association between early-life sugar rationing and lower cardiovascular risk in adulthood further illustrates the potential long-term relevance of early dietary exposures.

This life-course perspective may nevertheless inform prevention across multiple stages of pediatric development. Prevention should not begin only after obesity or metabolic dysfunction has developed. The pediatric window for intervention spans infancy and complementary feeding, when healthy beverage norms are established, through the years of supporting families, modifying school environments, counseling adolescents, and implementing population-level measures that reduce the availability, affordability, and promotion of high-sugar beverages. Importantly, the effects of structural policies should not be assumed to be uniform across pediatric populations: evaluations such as that of the UK SDIL indicate meaningful heterogeneity by age and sex, including subgroups in which no reduction in obesity was detected. The effect sizes attached to individual interventions are generally modest and heterogeneous, supporting a layered prevention strategy rather than reliance on any single measure. Viewing SSB consumption through a life-course lens therefore shifts attention from the late correction of established adolescent behavior toward the earlier development of healthier beverage environments. This perspective provides a framework for integrating preventive opportunities across childhood, while its individual components and proposed longitudinal pathways require further evaluation in prospective and intervention studies.

## Figures and Tables

**Table 1:** Key studies informing the life-course model of sugar-sweetened beverage exposure and pediatric adiposity.

Study [Ref]	Design	Population	Main Finding as Reported in the Source	Key Limitation and Interpretive Confidence
Müller 2023 [10]	Prospective birth cohort (OPALINE)	312 infants, 3–12 months	No association between sweetness exposure and sweetness liking at 6 or 12 months, despite a sharp rise in exposure; liking at 6 months predicted liking at 12 months.	Moderate confidence for sweet-preference outcomes; not direct evidence for SSB-related adiposity.
Baxter 2022 [11]	Scoping review	131 studies; families with children 0–5 y experiencing food insecurity or disadvantage	Interventions promoting responsive feeding may not meet the needs of these households; design should be sensitive to poverty-related constraints.	Low confidence for intervention effectiveness.
Hu 2023 [12]	Cross-sectional, pooled national school surveys	405,528 adolescents, 107 countries and regions	Country-level partial correlation 0.44 (95% CI 0.26–0.58; *p* < 0.001); individual-level OR 1.14 (1.08–1.21) for daily vs. non-daily consumption.	Low confidence for causal inference.
Abbasalizad Farhangi 2022 [13]	Dose–response meta-analysis of observational studies	33 studies, 121,282 children and adolescents	High vs. low SSB intake: BMI +0.75 kg/m^2^ (0.35–1.15); waist +2.35 cm (1.34–3.37); body fat +2.81 pp (2.21–3.41). No departure from linearity.	Moderate confidence for association; low confidence for causality.
Wu 2021 [14]	Prospective cohort with DXA (Project Viva)	783 children, exposure at 1 y, outcomes at 7.8 and 13 y	Each 120 mL/day of fruit juice at 1 y: visceral adipose tissue area SDS β = 0.08 (0.03–0.13); weaker for subcutaneous fat (β = 0.05).	Moderate confidence for fruit-juice exposure; indirect for SSBs.
Harnois-Leblanc 2026 [15]	Longitudinal marginal structural models (Project Viva)	972 children followed to age 17 y	Limiting SSBs to 1 serving/week across childhood: HOMA-IR −0.28 units (−0.61 to 0.02); waist −1.91 cm (−3.79 to −0.05) in males. Estimates near null in females.	Moderate confidence.
Nguyen 2026 [16]	Prospective cohort, up to 25 y (Growing Up Today Study)	25,749 participants, median age 12 → 36 y	≥2 SSB servings/day vs. <3/week: 52% higher risk of incident hypertension. Substituting one daily SSB serving with whole fruit: ~22% lower risk. Total fructose intake was not associated with risk.	Moderate confidence.
Taillie 2021 [17]	Before-and-after study of household purchases	2381 Chilean households, 2015–2017	After Phase 1 of the Chilean labeling law: overall calories purchased −3.5%; sugar purchased −10.2% relative to counterfactual.	Moderate confidence for purchasing outcomes.
Andreyeva 2022 [18]	Systematic review and meta-analysis of implemented SSB taxes	86 studies; 62 contributed to meta-analysis	Tax pass-through 82% (66–98%); SSB sales −15% (−20% to −9%); no evidence of substitution to untaxed beverages.	Moderate confidence for price and sales outcomes.
Rogers 2023 [19]	Interrupted time series, National Child Measurement Programme	Over 1 million English children measured annually	19 months after the UK levy, year-6 girls: obesity prevalence −1.6 percentage points (1.1–2.1), an 8.0% relative reduction; greatest in the two most deprived quintiles. No change in year-6 boys or reception children.	Moderate confidence.

Abbreviations: BMI, body mass index; CI, confidence interval; DXA, dual-energy X-ray absorptiometry; HOMA-IR, homeostatic model assessment for insulin resistance; OR, odds ratio; pp, percentage points; SDS, standard deviation score; SSB, sugar-sweetened beverage.

## Data Availability

No new data were created or analyzed in this study. Data sharing is not applicable to this article.

## Abbreviations

The following abbreviations are used in this manuscript:

AMP	adenosine monophosphate
AMPK	AMP-activated protein kinase
ATP	adenosine triphosphate
BMI	body mass index
CI	confidence interval
DXA	dual-energy X-ray absorptiometry
HeLTI	Healthy Life Trajectories Initiative
HOMA-IR	homeostatic model assessment for insulin resistance
HR	hazard ratio
LDL	low-density lipoprotein
MASH	metabolic dysfunction-associated steatohepatitis
MASLD	metabolic dysfunction-associated steatotic liver disease
OR	odds ratio
SDIL	Soft Drinks Industry Levy
SSB	sugar-sweetened beverage
